# Tuning Parameters for Polygenic Risk Score Methods Using GWAS Summary Statistics from Training Data

**DOI:** 10.21203/rs.3.rs-2939390/v1

**Published:** 2023-05-31

**Authors:** Wei Jiang, Ling Chen, Matthew J. Girgenti, Hongyu Zhao

**Affiliations:** 1 Department of Biostatistics, Yale School of Public Health, New Haven, CT, USA.; 2 Department of Statistics, Columbia University, New York, NY, USA.; 3 Department of Psychiatry, Yale School of Medicine, New Haven, CT, USA.

## Abstract

Predicting genetic risks for common diseases may improve their prevention and early treatment. In recent years, various additive-model-based polygenic risk scores (PRS) methods have been proposed to combine the estimated effects of single nucleotide polymorphisms (SNPs) using data collected from genome-wide association studies (GWAS). Some of these methods require access to another external individual-level GWAS dataset to tune the hyperparameters, which can be difficult because of privacy and security-related concerns. Additionally, leaving out partial data for hyperparameter tuning can reduce the predictive accuracy of the constructed PRS model.

In this article, we propose a novel method, called PRStuning, to automatically tune hyperparameters for different PRS methods using only GWAS summary statistics from the training data. The core idea is to first predict the performance of the PRS method with different parameter values, and then select the parameters with the best prediction performance. Because directly using the effects observed from the training data tends to overestimate the performance in the testing data (a phenomenon known as overfitting), we adopt an empirical Bayes approach to shrinking the predicted performance in accordance with the estimated genetic architecture of the disease. Results from extensive simulations and real data applications demonstrate that PRStuning can accurately predict the PRS performance across PRS methods and parameters, and it can help select the best-performing parameters.

## 1. Introduction

The advent of genome-wide association studies (GWAS) has led to the discovery of numerous loci associated with most common diseases [1]. These discoveries also provide the opportunity for predicting risks from an individual’s genotypes [2]. Accurate genetic risk prediction can enable us to identify high risk individuals and facilitate disease prevention and early treatment [3].

Polygenic risk score (PRS) is commonly used in genetic risk prediction due to its simplicity and resulting from additive assumption. Both empirical and theoretical studies have shown that the additive component is expected to account for most of the genetic variance of complex traits [4]. Based on this additive assumption, PRS sums the allele dosages of single nucleotide polymorphisms (SNPs) weighted by their estimated effect sizes [5].

Various PRS methods have been proposed to estimate the effect sizes of SNPs from a GWAS dataset. Compared to individual-level genotype data, summary statistics are more accessible without security and privacy concerns [6][7]. Many PRS methods proposed recently estimate SNP effects with GWAS summary statistics. One of the simplest is clumping and thresholding (C+T)[8][9][10][11][12][13][14], in which linkage disequilibrium (LD) clumping is applied to the SNPs that pass a *p*-value threshold. Another related method is pruning and thresholding (P+T), which only includes the SNPs whose *p*-values exceed a threshold after LD pruning. Both LD clumping and LD pruning are step-wise heuristic procedures that select a set of approximately independent SNPs. Compared to LD pruning, LD clumping selects the independent SNPs after *p*-value thresholding. Therefore, SNPs showing stronger associations with the disease are preserved, which is preferred in constructing PRS. We note that some literature referred to C+T as P+T, but we treat them as distinct methods in our following discussion.

It is important to note that for both C+T and P+T, only a portion of independent SNPs are utilized in constructing the PRS model, while other SNPs and LD information are ignored. To further improve the prediction accuracy of genetic risks, many PRS methods have been proposed to incorporate genome-wide SNPs and their LD information, such as LDpred [15], sBayesR [16], PRS-CS [17] and SDPR [18]. LDpred imposes a point-normal prior for the SNP effect sizes and infers the posterior mean effect sizes using a Markov Chain Monte-Carlo (MCMC) procedure. To allow more general effect size distributions, sBayesR performs Bayesian posterior inference based on a mixture prior of point and three normal distributions that represent SNPs with zero, small, medium, and large effects respectively. SDPR performs Bayesian posterior inference based on a Dirichlet process modeling effect sizes with a mixture of 1000 normal distributions. To reduce the computational burden from the combination of different components in millions of SNPs, PRS-CS places a continuous shrinkage prior to the SNP effect sizes in a Bayesian framework. All these LD-based methods have demonstrated their superior performance in some datasets of complex diseases. However, none of them has a dominant performance over other methods.

Among these PRS methods, P+T, C+T, and LDpred rely on hyperparameters that need to be specified by users beforehand. Although PRS-CS and sBayesR have options to estimate hyperparameters with an additional layer of prior distributions, users can also specify the hyperparameters themselves. For all PRS methods that require tuning hyperparameters, an external individual-level genotype dataset is needed to evaluate different parameter values and choose the best-performing ones. However, as we mentioned before, individual-level genotype data are less accessible than summary statistics. Besides, it is not efficient to leave out a portion of data just for tuning parameters and to estimate SNP effects with the remaining data, leading to information loss and reduced performance for PRS methods. These concerns motivated us to develop a method that can evaluate the performance of a PRS model based on summary statistics used for model training.

For diseases with a binary phenotype, the area under the receiver operating characteristic (ROC) curve (AUC) is the most commonly used criterion in practice for evaluating PRS [5][19][20]. In 2018, Song et al. [21] proposed an estimator of AUC using only summary statistics. This method makes use of an equivalent definition of AUC, i.e. the probability of a PRS from a random case being larger than a PRS from a random control. Based on this definition, AUC can be approximated by a function of the GWAS summary statistics. This method can tune the hyperparameters of a PRS model with summary statistics from another GWAS.

To maximize the power of identifying loci associated with common diseases, some large consortia have conducted meta-analyses of all accessible studies and released summary statistics from these meta-analyses. These summary statistics are usually used as training data to optimize the prediction power of PRS models. In this situation, it is difficult to gain access to summary statistics from another independent GWAS. This problem can not be well addressed if we simply plug the summary statistics from the training data into the derived AUC function, because the variants with larger effects tend to have their effect sizes overestimated and these variants have a larger influence on the PRS than the variants exhibiting small effects. This phenomenon is known as overfitting [22]. If we use the observed effects directly, the overfitting would lead to an inflated predicted value of the AUC and the incorrectly selected values of the hyperparameters.

Built on Song’s method, we propose PRStuning, a method that requires only summary statistics from the training data to predict the conventional AUC that needs to be evaluated on another individual-level genotype dataset. We incorporate empirical Bayes (EB) theory to shrink the effect sizes of SNPs, which leads to the attenuation of the predicted AUC so as to overcome the overfitting phenomenon [23]. In PRStuning, we adopt a point-normal mixture model as the prior distribution of SNP effects and estimate the parameters in the model with GWAS summary statistics from the training data. There are two settings depending on the dependency across the selected SNPs used for training the PRS model. When the SNPs are independent, e.g., the SNPs used in P+T, we utilize an expectation-maximization (EM) algorithm to estimate the parameters in the prior distribution and calculate the posterior distribution of the AUC based on a closed-form formula. When SNPs are dependent due to LD, we use a Gibbs-sampling-based State-Augmentation for Marginal Estimation (SAME) algorithm [24] to estimate the parameters in the model and obtain the Monte-Carlo (MC) samples of the predicted AUC. Once this is accomplished, we can select the parameter values for the PRS method with the best predicted AUC.

We applied PRStuning to GWAS datasets of three common diseases, including coronary artery disease (CAD), type 2 diabetes (T2D), and inflammatory bowel disease (IBD), with three PRS methods, namely P+T,C+T, and LDPred. Results from extensive simulations and real data applications demonstrate that PRStuning can accurately predict the PRS performance across PRS methods and parameters, and it can help with parameter selections.

## 2 Results

### 2.1 Overview of PRStuning

Define $g_{i,m}\in \{0,\;1,\;2\}$ as the genotype score of SNP m for individual i. A PRS for individual i is the sum of the genotypes $g_{i}=\left(g_{i,1},\ldots ,g_{i,M}\right)$ weighted by the corresponding effects $\omega =\left(\omega_{1},\ldots ,\omega_{M}\right)$, i.e.,

(1) $$PRS_{i}=\sum_{m=1}^{M}\omega_{m}g_{i,m}.$$

Here M is the total number of pre-selected SNPs used for constructing PRS. Please note that not all SNPs collected in the training GWAS data are necessarily used in PRS calculation. Some PRS methods incorporate SNP selection steps based on the associations of the SNPs with the disease. Here we define the pre-selected SNPs as the SNPs used in building the PRS model before running any selection step. For example, the pre-selected SNPs in C+T are actually genome-wide SNPs collected in the training GWAS data and the LD clumping procedure used in C+T is a selection step based on the observed association strength. In contrast, LD pruning is a selection step unrelated to SNP associations with the disease. Therefore, the pre-selected SNPs in P+T are the SNPs selected after an LD pruning step. Different PRS methods have been proposed to estimate the weight vector ω from a GWAS dataset or its summary statistics for the disease of interest. Here and after we regard ω as the inferred values from the PRS method of interest.

Based on the definition of AUC and the distribution of PRS, Song [21] formulated AUC as

(2) $$AUC=\Phi \left(\Delta \right),$$

Where

(3) $$\Delta :=\frac{2\sum_{m=1}^{M}\omega_{m}\delta_{m}}{\sqrt{\tau_{0}^{2}+\tau_{1}^{2}}}\;\text{and}\;\tau_{j}^{2}=\sum_{m=1}^{M}\omega_{m}^{2}s_{j,m}^{2}+2\sum_{m_{1}<m_{2}}\omega_{m_{1}}\omega_{m_{2}}R_{m_{1},m_{2}}s_{j,m_{1}}s_{j,m_{2}},$$

where j=0 indicates controls and 1 indicates cases. Here for SNP m, we use $f_{j,m}$ to denote the frequency of the reference allele, $s_{j,m}^{2}\;:=2f_{j,m}\left(1-f_{j,m}\right)$ to denote the variance of the genotype, and $\delta_{m}\;:=f_{1,m}-f_{0,m}$ records the difference between the allele frequencies of the cases and controls, and Φ(⋅) is the cumulative distribution function of a standard normal distribution. We use $R_{m_{1},m_{2}}$ to denote the LD coefficient between SNP $m_{1}$ and SNP $m_{2}$.

We can calculate $\tau_{j}^{2}(j=0,\;1)$ by directly plugging in the observed values of allele frequencies and LD coefficients since $\tau_{j}^{2}$ is not directly related to the SNPs’ effects on the disease. The observed allele frequencies can be obtained from summary statistics of the GWAS, and LD information can be extracted from another genotype dataset. Some large projects such as the 1000 Genomes project (1KG) [25] and the HapMap3 project (HM3) [26] have made their data publicly available and we can use them as reference panels to calculate the LD coefficients.

For $\delta_{m}$ in Eq. (3), if we directly plug in the observed allele frequencies ${\overset{ˆ}{f}}_{0,m}$ and ${\overset{ˆ}{f}}_{1,m}$ from GWAS, the SNPs exhibiting large allele frequency differences tend to have their effect sizes overestimated, and these SNPs have larger contributions to the PRS than the SNPs showing smaller effects. The overfitting of the SNP effects would lead to an inflated predicted value of the AUC and incorrectly selected values of the hyperparameters. Therefore, we adopt an Empirical Bayes method in PRStuning to shrink the effects so as to reduce the influence of overfitting.

In GWAS, z-scores from the allele frequency difference test are usually used to assess the association of each SNP with the disease. Each z-score is calculated with the following formula:

(4) $$z_{m}=\frac{{\overset{ˆ}{f}}_{1,m}-{\overset{ˆ}{f}}_{0,m}}{\sqrt{s_{1,m}^{2}/4n_{1}+s_{0,m}^{2}/4n_{0}}},$$

where ${\overset{ˆ}{f}}_{j,m}$ is the observed allele frequency for each group, $s_{j,m}^{2}$ is the variance of the genotype in the controls or cases, and $n_{0}$, $n_{1}$ are the sample sizes of the two groups. To simplify this expression, we define $s_{m}\;:=\sqrt{s_{1,m}^{2}/4n_{1}+s_{0,m}^{2}/4n_{0}}$. Based on this definition, we have $z_{m}|\delta_{m}∼N\left(\delta_{m}/s_{m},\;1\right)$ given the allele frequency difference $\delta_{m}$.

We will further demonstrate in the Supplementary Note that $\delta =\left(\delta_{1},\ldots ,\delta_{M}\right)$ is related to the LD pattern among the pre-selected SNPs and the underlying effects of the risk SNPs in terms of the allele frequency differences between the two groups, i.e.,

(5) $$\delta =SRS^{-1}\beta ,$$

where S is a diagonal matrix with the m-th diagonal element equal to $s_{m},\;\beta =\left(\beta_{1},\ldots ,\beta_{M}\right)$ with $\beta_{m}$ being the underlying effect of SNP m, and R is the LD coefficient matrix. Given δ, the joint distribution of the z-scores $z=\left(z_{1},\ldots ,z_{M}\right)$ is

(6) $$z|\delta ∼N\left(S^{-1}\delta ,\;R\right).$$

We further assume that the standardized effect $\beta_{m}/s_{m}$ follows a point-normal distribution, i.e.,

(7) $$\frac{\beta_{m}}{s_{m}}\overset{iid}{∼}\left(1-\pi \right)\delta_{0}+\pi N\left(0,\sigma^{2}\right).$$

Here $\delta_{0}$ is a point mass at zero, π represents the prior proportion of SNPs that have an effect on the disease, and $\sigma^{2}$ is the variance of $\beta_{m}/s_{m}$ in the risk SNPs. This point-normal distribution is also used in LDpred as the prior distribution. With this assumption, we derived an expectation-maximization (EM) algorithm to estimate $\left(\pi ,\sigma^{2}\right)$ and calculated the posterior distribution of the AUC when pre-selected SNPs are independent. When SNPs are linked by LD, we derived a Gibbs-sampling-based SAME algorithm to estimate $\left(\pi ,\sigma^{2}\right)$ and obtained the MC samples of the predicted AUC. Once this is accomplished, we can select the parameter values for the PRS method with the best predicted AUC. Details of PRStuning are presented in Section 4.

### 2.2 Simulation experiments

For our simulation experiments, we considered predicting the performance and tuning the parameters for three commonly used PRS methods, namely, P+T,C+T, and LDpred. In the experiments, we varied the *p*-value thresholds for P+T and C+T from {1,5e–1,5e–2,5e–3,5e–4,5e–5,5e–6}. While for LDpred, we chose the proportion of the risk SNPs π from {1,3e–1,1e–1,3e–2,1e–2,3e–3,1e–3,3e–4,1e–4,3e–5,1e–5}. These are the default settings for the three PRS methods.

There are two purposes of our method: to predict the AUC and to select tuning parameters. In our experiments, we used another independent dataset with individual-level genotype data as testing data. The AUC of the PRS assessed on the testing data and the parameters showing the best prediction performances on the testing data were treated as benchmarks. To evaluate the performance of PRStuning, we evaluated the performance of PRStuning with two measures: the correlation of the AUC estimates $\left(\rho_{AUC}\right)$ and the relative difference of the highest AUC estimates $\left(rd_{AUC}\right)$. We define $\rho_{AUC}$ as the correlation of the PRStuning-predicted AUC values and those estimated on the testing data. A high value of $\rho_{AUC}$ indicates that the predicted AUC using our method is highly correlated with the AUC on the testing data. We define $rd_{AUC}$ as the relative difference between the predicted AUC with the best-performing parameter tuned by PRStuning and the AUC with the bestperforming parameters on the testing data. Here best-performing parameters are defined as those achieving the highest AUC values. A small value of $rd_{AUC}$ indicates that the tuning parameter selected by PRStuning and the actual best-performing parameter have comparable performances. These two metrics are complementary to each other in the sense that, $\rho_{AUC}$ measures how much the AUC patterns across parameter values for PRStuning and testing data align with each other, while $rd_{AUC}$ measures the point difference between the highest AUC values for the two methods. Therefore, we would like to evaluate the results with both metrics.

#### 2.2.1 Independent SNPs for constructing PRS

We first consider the case where the pre-selected SNPs are independent. In our simulations, we set the prevalence of the disease to κ=1%. For each SNP, we simulated its allele frequency in the general population based on a uniform distribution U(0.05,0.95). Then we generated its risk effects on the disease based on the two-component mixture model Eq. (7), in which we set the proportion of the risk SNPs to π=0.05 and the variance of the risk effects to $\sigma^{2}=0.001n$. Here n is the total sample size of the GWAS used in the training data. We assume the GWAS is balanced with an equal number of cases and controls. According to the central limit theorem, we have $s_{m}\propto 1/\sqrt{n}$. Hence it is reasonable to assume $\sigma^{2}\propto n$.

In total, we simulated M=10,000 independent SNPs and varied the sample size from 4, 000 to 10, 000 in the training GWAS to explore the performance trend across different sample sizes. Each sample size setting was replicated 50 times. And for each replication, we simulated additional 1000 cases and 1000 controls as testing data.

We used the AUC evaluated on the testing data as the benchmark, and compared the AUC predicted by PRStuning and the unadjusted AUC obtained by directly plugging in the training summary statistics with the benchmark. Since all SNPs are independent, we only considered P+T as the PRS method.

**Fig. 1:**
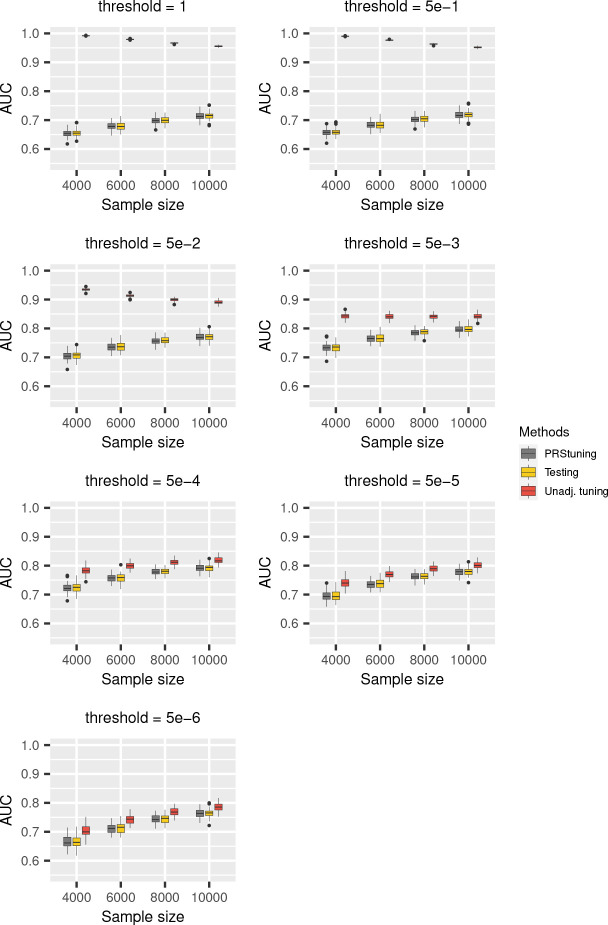
AUC boxplots for P+T in the simulation experiments with independent SNPs. We changed the *p*-value threshold from {1, 5e–1, 5e–2, 5e–3, 5e–4, 5e–5, 5e–6} and the sample sizes of training data from 4,000 to 10,000. The grey, yellow, and red panels represent AUC predicted from PRStuning, AUC evaluated on testing data, and the unadjusted AUC directly estimated by plugging in the training summary statistics, respectively. The AUC evaluated on the testing data is the benchmark. PRStuning is able to yield AUC estimates comparable to the benchmark results.

Figure 1 shows the boxplots of AUC values corresponding to different *p*-value thresholds and sample sizes of training data for P+T. The grey, yellow, and red panels represent AUC predicted from PRStuning, AUC calculated from testing data, and the unadjusted AUC obtained by directly plugging in the training summary statistics, respectively. As expected, the unadjusted AUC estimates were inflated compared to the benchmark due to the overfitting problem. In contrast, with the same summary statistics from the training data, PRStuning was able to shrink the estimates of allele frequency differences and produce AUC estimates comparable to those from the testing data.

In order to further demonstrate the accuracy of PRStuning, we summarize the average correlation of the AUC estimates $\rho_{AUC}$ and the average relative difference of the best-performing AUC estimates $rd_{AUC}$ in Table 1. Those metrics are complementary to each other since two vectors can be perfectly correlated but differed a lot. The values of $\rho_{\text{AUC}}$ were at least 0.976, which indicates that PRStuning is capable of accurately predicting the AUC pattern on testing data. Moreover, the average values of $rd_{AUC}$ were at most 1.3%, indicating that PRStuning can effectively select parameter values that achieve performance comparable to the best-performing parameter in the testing data. Note that $\rho_{\text{AUC}}$ increased and $rd_{\text{AUC}}$ decreased as the sample size of training GWAS increased. This is expected because a larger sample size in the training data can lead to higher accuracy in estimating allele frequency differences.

#### 2.2.2 Correlated SNPs for constructing PRS

In this section, we considered the case where the pre-selected SNPs are not filtered by any independence criterion for SNPs. In this case, the pre-selected SNPs are linked as reflected in their LD.

We first performed simulations with SNPs with an AR(1) auto-regressive LD structure. We fixed the auto-regressive coefficient ρ to 0.2, which is the correlation coefficient between two adjacent SNPs. Similar to the simulation scenario with independent SNPs, we simulated the reference allele frequencies in the population from U(0.05,0.95), and the risk effects from a point normal distribution Eq. (7), in which π=0.05 and $\sigma^{2}=0.0005n$. The variance of risk effects is proportional to the sample size of the GWAS since $s_{m}\propto 1/\sqrt{n}$ according to the central limit theorem.

We varied the sample size from 4,000 to 10,0000 in the training GWAS and generated 20 replications for each sample size. We used CorBin [27], an R package for generating high dimensional binary data with a specific correlation structure, to generate individual-level genotype data. Specifically, we generated 1000 cases and 1000 controls as testing data for each replication. We also simulated additional 1000 samples as a reference panel for calculating LD coefficients. We used both C+T and LDpred as the PRS methods in this experiment, and the LD radius was set to 5 for both methods.

**Fig. 2:**
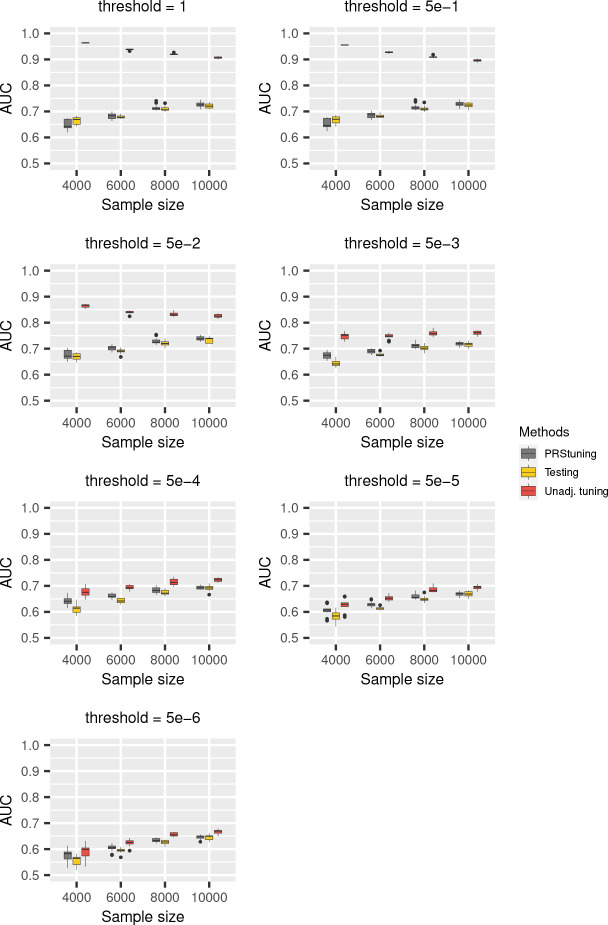
AUC boxplots for C+T in the simulation experiments with correlated SNPs. We changed the *p*-value threshold from {1, 5e–1, 5e–2, 5e–3, 5e–4, 5e–5, 5e-6} and the sample sizes of training data from 4,000 to 10,000. The grey, yellow, and red panels represent AUC predicted from PRStuning, AUC evaluated on testing data, and the unadjusted AUC directly estimated by plugging in the training summary statistics, respectively. The AUC evaluated on the testing data is the benchmark. PRStuning is able to yield AUC estimates comparable to the benchmark results.

**Fig. 3:**
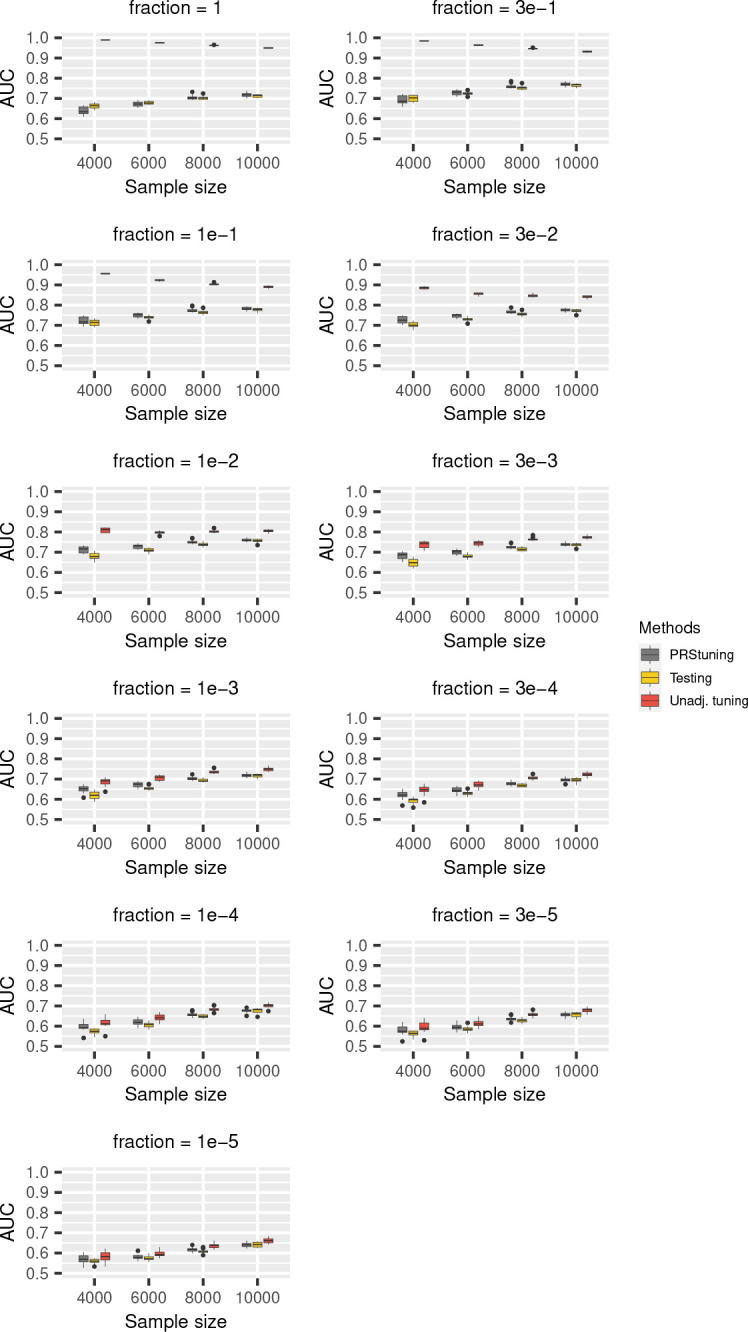
AUC boxplots for LDpred in simulation experiments with correlated SNPs. We changed the proportion of risk SNPs from {1, 3e–1, 1e–1, 3e–2, 1e–2, 3e–3, 1e–3, 3e–4, 1e–4, 3e–5, 1e–5} and the sample sizes of training data from 4,000 to 10,000. The grey, yellow, and red panels represent AUC predicted from PRStuning, AUC calculated from testing data, and the unadjusted AUC, respectively.

To demonstrate the predictive accuracy of PRStuning, we again regarded the AUC evaluated on the testing data as the benchmark and compared the AUC predicted by PRStuning and the unadjusted AUC with the benchmark. Figures 2 and 3 demonstrate the AUC boxplots for C+T and LDpred with different parameter values, respectively. For both PRS methods, the unadjusted AUC estimates were largely overestimated compared to the benchmark due to overfitting. On the contrary, the AUC estimates predicted by PRStuning were very close to the benchmark results, especially when the sample size became large.

We summarize the average values of $\rho_{AUC}$ and $rd_{AUC}$ in Tables 2 and 3. For both C+T and LDpred, the values of $\rho_{AUC}$ were at least 0.852 in all sample size settings, indicating PRStuning can accurately predict the AUC on testing data. The average values of $rd_{AUC}$ were below 2%, meaning PRStuning can effectively select a parameter that achieves performance comparable to the actual best-performing parameter on the testing data. Again, we can observe an increasing tendency in $\rho_{AUC}$ and a decreasing tendency in $rd_{AUC}$ as we increase the sample size of the training GWAS as the result of the increase in estimation accuracy of the allele frequency differences.

We further evaluated PRStuning with simulations based on real genotype data. The experiments were conducted based on genotype data collected from the UK Biobank (UKBB) [28], which collected genetic and health records from around 500, 000 participants in the UK. The quality control procedure is sum marized in the Supplementary Note. We only selected independent individuals with European ancestry in the experiments. Since only SNPs presented in the HapMap 3 project (HM3 SNPs) were used in the reference panel for reliable LD estimation and computation efficiency, we focused on the SNPs in HM3 in the UKBB dataset. This resulted in a total of 1, 027, 699 HM3 SNPs and 272, 751 individuals passing the quality control criteria.

We used the two-component mixture model Eq. (7) to simulate risk effects for SNPs with π=0.1% and $\sigma^{2}=0.04$. The phenotypes of the individuals were simulated based on the additive assumption. Among all individuals, we randomly selected 80% of them for GWAS analysis to calculate the summary statistics as training data and the rest as testing data. We used the data collected from the 1000 Genomes Project (1KG) [25] as the reference panel for calculating LD. In the experiments, we used both C+T and LDpred as the PRS methods and compared the AUC estimates predicted by PRStuning with the values calculated on the testing data.

In Table 4, we summarize the AUC results of C+T with different parameter values for both PRStuning and testing genotype data. The AUC estimates from PRStuning were very close to the actual AUC values obtained from the testing data. The correlation $\rho_{\text{AUC}}$ reached 0.996 and the relative difference $rd_{\text{AUC}}$ was 3.7%. Table 5 shows the AUC results for LDpred with different parameter values. Similarly, we achieved excellent prediction with the actual AUC values from the testing data. The $\rho_{\text{AUC}}$ reached 0.998 and $rd_{\text{AUC}}$ was just 1.3%. It is worth noting that PRStuning was able to detect the dramatic decrease in the testing performance of LDpred when π was dropped from 1e–1 to 3e–2. These results further suggest the accuracy in AUC estimation and effectiveness in parameter tuning using PRStuning on SNPs linked by LD.

### 2.3 Real data applications

We applied PRStuning to GWAS summary statistics from three diseases, including coronary artery disease (CAD), type 2 diabetes (T2D), and inflammatory bowel disease (IBD). Table 6 summarizes the sources of the publicly available GWAS summary statistics and their corresponding sample sizes. Note that the summary statistics from all three datasets are results of meta-analyses and the reported sample sizes represent the total numbers of individuals among all aggregated studies. The actual sample size used to calculate the summary statistics of each SNP was less than the reported sample size, since some of the studies may not have genotypes on this SNP.

We used these summary statistics to train the PRS models based on P+T, C+T, and LDpred. Then we used the data collected from the UKBB as the testing data for evaluating the actual prediction performance of the built PRS models. Only the SNPs with minor allele frequencies greater than 5% were included in building the PRS models. Details of the quality control procedure and phenotype extraction method for the UKBB data are provided in the Supplementary Note.

**Fig. 4:**
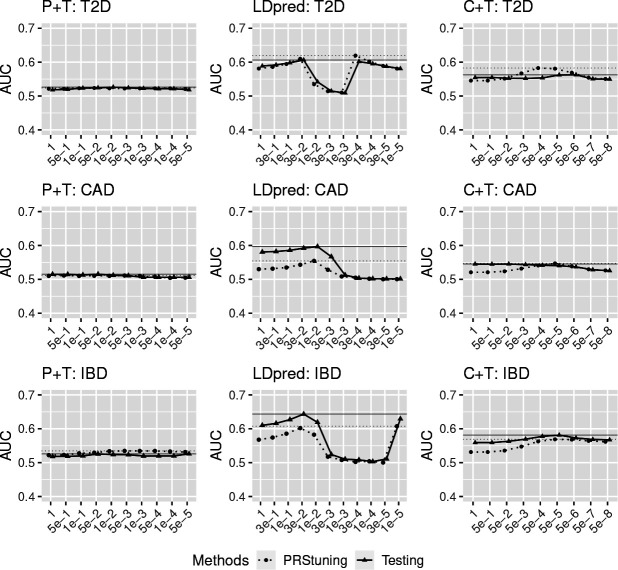
The predicted AUC by PRStuning and the actual AUC on testing data for three diseases with PRS models built from P+T, LDpred, and C+T. The left, middle, and right panels present the results of P+T, LDpred, and C+T, respectively. The dotted and solid horizontal lines refer to the highest AUC for PRStuning and testing data. The overall patterns of AUC predicted by PRStuning and calculated from testing data across different parameter values were similar.

In PRStuning, we adopted the EM algorithm 1 for PRS models built by P+T since the pre-selected SNPs were approximately independent, and the Gibbs sampling-based SAME algorithm 2 for C+T and LDpred due to the presence of LD among the pre-selected SNPs. Figure 4 shows the predicted AUC by PRStuning and the actual AUC on testing data for three diseases with different PRS models. The dotted and solid horizontal lines respectively refer to the highest AUC for PRStuning and testing data. It is evident in the figure that the AUC predicted by PRStuning and the AUC calculated from testing data had similar patterns across different parameter values, particularly for LDpred. For CAD, the AUC of LDpred increased when the risk SNP proportion π was reduced from 1 to 1e – 2. It peaked at 1e – 2 and then started to decrease when we kept reducing the value of π. This pattern was exactly predicted by PRStuning. More complex patterns of AUC were observed for LDpred in T2D and CAD. The AUC values in both diseases had double modes across parameter values. For T2D, the AUC of LDpred peaked at 3e – 2 and 3e – 4. For IBD, the AUC of LDpred peaked at 3e – 2 and 1e – 5. Still, PRStuning predicted the exact same patterns of AUC for both diseases. This demonstrates the high predictive accuracy of PRStuning.

To further explain why there were double modes for AUC with different parameter values, we refer back to the calculation of Δ in Eq. (3) since AUC is monotonically increasing with respect to Δ. The numerator of Δ is a linear combination of the weights $\omega ={\left(\omega_{1},\ldots ,\omega_{M}\right)}^{T}$ used in PRS, whereas the denominator is the square root of a quadratic function of ω, which can be further expressed as

(8) $$\sqrt{\tau_{0}^{2}+\tau_{1}^{2}}=\sqrt{\omega^{T}\left(S_{0}RS_{0}+S_{1}RS_{1}\right)\omega ,}$$

where $S_{0}$ and $S_{1}$ are diagonal matrices with diagonal elements encoding $\left(s_{0,1},\ldots ,s_{0,M}\right)$ and $\left(s_{1,1},\ldots ,s_{1,M}\right)$, respectively. The weights in the PRS model were calculated based on different values of parameters. In Supplementary Figure 1, we demonstrate the denominators and numerators of Δ with different parameter values in LDpred for the three diseases. From the figure, we can observe that both the denominator and numerator were actually unimodal functions with respect to the parameter values that peak at different parameter values. Their ratio led the Δ to become bimodal functions with respect to the parameter values.

In Figure 4, we do observe some underestimation of AUC for LDpred and C+T on CAD and IBD. This is because the summary statistics collected are results of meta-analyses. The actual sample size used for calculating the summary statistics of each SNP is less than the reported sample size, because some of the studies may not have genotypes at this SNP. Some consortia, such as GLGC [32], provide the sample size used for calculating summary statistics of each SNP, but most consortia do not provide this information. Even if we have the sample size for each SNP, we can not infer the number of non-overlapping individuals for calculating summary statistics of two SNPs. The non-overlapping individuals will change the correlations between *z*-values. In our analysis, we simply plugged the total sample sizes reported by the summary statistics into PRStuning. According to Eq. (16), the inflation of the sample size would lead to the systematic underestimation of $s_{m}$. Based on Eq. (2), we know that AUC is monotonically increasing with respect to Δ, and we have $\Delta \propto \sum_{m=1}^{M}\omega_{m}\delta_{m}$ and $\delta_{m}=\beta_{m}s_{m}$. Therefore, the underestimation of $s_{m}$ would further lead to the underestimation of AUC.

To further illustrate the predictive accuracy of PRStuning, we calculated $\rho_{AUC}$ and $rd_{AUC}$ with different PRS methods for the three diseases. The results of $\rho_{AUC}$ and $rd_{AUC}$ are summarized in Table 7. The low values of $rd_{AUC}$ indicate that the prediction performance under the PRStuning-selected parameter approximated the best performance on the testing data accurately, especially for C+T and P+T. Even though LDpred had higher $rd_{AUC}$ compared to the other two PRS methods, it yielded values of $\rho_{AUC}$ all above 0.96. The high values of $\rho_{AUC}$ indicate that PRStuning can accurately predict the pattern of AUC with respect to the parameters on the testing data. This can be clearly observed from Figure 4. These results show that PRStuning can help us select the best-performing parameters in PRS methods with only summary statistics from the training data.

We note that the correlation between AUC predicted by PRStuning and calculated from the testing data was negative with C+T for CAD. However, also note that the standard deviations among the AUC values with different parameters for both methods were less than 0.01 in this scenario. The extremely small standard deviations of AUC contribute to the large variation of the correlation. Therefore, the correlation is relatively uninformative in characterizing the relationship between the predicted and the actual AUC values. On the other hand, the small value of $rd_{AUC}$ (0.4%) suggests the effectiveness of PRStuning.

## 3. Discussion

PRS methods have proven useful for the prediction of common disease risks, which can help improve disease prevention and early treatment. Some PRS methods require users to specify the values for hyperparameters. However, to tune the hyperparameters, an external individual-level genotype dataset is often needed to evaluate the prediction performance of different hyperparameter values. However, individual-level genotype data are much less accessible compared to GWAS summary statistics due to privacy and security concerns. Additionally, leaving out partial data for hyperparameter tuning can also reduce the predictive accuracy of the PRS model.

These concerns motivated us to propose PRStuning, an empirical Bayes method that only requires summary statistics from the training GWAS to evaluate PRS and tune the parameters. PRStuning is based on an AUC estimator proposed in [21], which is a function of the GWAS summary statistics. However, plugging the training summary data directly into this estimator would cause overfitting, leading to an inflation of the predicted AUC. To tackle this problem, we adopted the empirical Bayes approach to shrinking the predicted AUC based on the estimated genetic architecture. Extensive simulation experiments and real data applications on three diseases with three PRS methods demonstrated that PRStuning is capable of accurately predicting the AUC on the testing data and selecting the best-performing parameters.

The core of PRStuning is to estimate the allele frequency differences among SNPs. To do so, we need to input the sample sizes of the cases and controls in the training data. Usually, they are provided in the sources of GWAS summary statistics. However, if the summary statistics were derived from a meta-analysis, not all SNPs were genotyped in all studies included in the meta-analysis. In this case, the actual sample sizes used for calculating the summary statistics are less than the reported total sample sizes in the meta-analysis for some SNPs. This may lead to underestimation in AUC according to Eq. (2). This phenomenon was observed when we applied PRStuning to C+T and LDpred on CAD and IBD, where the AUC estimates from PRStuning were lower than the actual values in the testing data. Nevertheless, according to our experimental results, the underestimation phenomenon will not influence the performance of parameter selection since the overall pattern of the AUC values with different parameter values can still be well-predicted by PRStuning.

Currently, we only considered tuning parameters for PRS methods on diseases or other binary phenotypes. For quantitative phenotypes, instead of AUC, predictive $r^{2}$ is commonly used as an evaluation criterion of the PRS model. Extending PRStuning to evaluating predictive $r^{2}$ and selecting hyperparameters on quantitative phenotypes is left as future work.

## 4 Method

### 4.1 Notations and assumptions

Based on the additive assumption, the PRS for individual i is the sum of the genotypes $g_{i}=\left(g_{i,1},\ldots ,g_{i,M}\right)$ weighted by the corresponding effects $\omega =\left(\omega_{1},\ldots ,\omega_{M}\right):$

(9) $$PRS_{i}=\sum_{m=1}^{M}\omega_{m}g_{i,m},$$

where M is the total number of the pre-selected SNPs used for constructing PRS. Depending on the specific PRS method, not all SNPs collected in the training GWAS data are necessarily used in PRS calculation. Some PRS methods incorporate steps for selecting SNPs based on their associations with the disease. Here we define the pre-selected SNPs as the SNPs used in building the PRS model before running a selection step. LD clumping is an example of the selection step based on the observed association strength. Hence, we refer to the pre-selected SNPs in C+T as genome-wide SNPs collected in the training GWAS data. On the contrary, LD pruning is a selection step unrelated to the associations of SNPs with the disease. Therefore, the pre-selected SNPs in P+T are the SNPs selected after an LD pruning step. Different PRS methods have been proposed to estimate the weight vector $\omega =\left(\omega_{1},\ldots ,\omega_{M}\right)$ from a GWAS dataset or its summary statistics for the disease of interest. Here and after we simply use ω to denote the effects already estimated from a PRS method.

Based on disease status, we divide individuals into the case and control groups. In the following, we use subscripts j=0 and j=1 to denote those from the control and case groups, respectively. For example, the frequency of the reference allele for SNP m among controls and cases are denoted as $f_{0,m}$ and $f_{1,m}$, respectively. The genotype $g_{i,m}$ of SNP m for an individual in the control group follows a binomial distribution $Bino\left(2,f_{0,m}\right)$ with mean $E\left[g_{0,m}\right]=2f_{0,m}$ and variance $s_{0,m}^{2}:=Var\left(g_{0,m}\right)=2f_{0,m}\left(1-f_{0,m}\right)$. Similarly, we have $g_{i,m}∼Bino\left(2,f_{1,m}\right)$ if the individual i is from the case group.

By the central limit theorem, PRS approximately follows a normal distribution in each group when the SNP number M is adequately large. For PRS methods involving SNP selection steps unrelated to the SNPs’ associations with the disease, such as P+T, M varies from ∼10 to ∼10K depending on the selection threshold. For PRS methods with genome-wide pre-selected SNPs, M ranges from ∼100K to ∼1M determined by the number of SNPs genotyped or imputed in the training data. Based on the central limit theorem, the PRS variables from the two groups follow normal distributions:

(10) $$PRS_{i}∼\left\{\begin{array}{ll} N\left(\eta_{0},\tau_{0}^{2}\right) & \text{if}\;i\in \text{control}\;\text{group}\\ N\left(\eta_{1},\tau_{1}^{2}\right) & \text{if}\;i\in \text{case}\;\text{group} \end{array}\right.,$$

where

(11) $$\eta_{j}=\sum_{m=1}^{M}2\omega_{m}f_{j,m},$$

and

(12) $$\tau_{j}^{2}=\sum_{m=1}^{M}\omega_{m}^{2}s_{j,m}^{2}+2\sum_{m_{1}<m_{2}}\omega_{m_{1}}\omega_{m_{2}}R_{m_{1},m_{2}}s_{j,m_{1}}s_{j,m_{2}},$$

for j=0 or 1. Here $R_{m_{1},m_{2}}$ corresponds to the correlation between SNP $m_{1}$ and SNP $m_{2}$, which is known as the LD coefficient.

For a binary phenotype, we usually use AUC as the criterion for evaluating the prediction performance of PRS. AUC is defined as the area under the ROC curve, which can also be calculated as the probability that a random PRS from the case group is larger than a random PRS from the control group [33]. Based on this fact and the distributions of PRS, Song, etc.[21] formulated AUC as

(13) $$AUC=\Phi \left(\Delta \right),$$

where

(14) $$\Delta :=\frac{\eta_{1}-\eta_{0}}{\sqrt{\tau_{0}^{2}+\tau_{1}^{2}}}=\frac{2\sum_{m=1}^{M}\omega_{m}\delta_{m}}{\sqrt{\tau_{0}^{2}+\tau_{1}^{2}}}.$$

Here $\delta_{m}\;:=f_{1,m}-f_{0,m}$ records the difference between the allele frequencies of the two groups for SNP m, and Φ(⋅) is the cumulative density function of a standard normal distribution.

To calculate $\tau_{0}^{2}$ and $\tau_{1}^{2}$ in Eq. (13), we can directly plug in the observed values of the allele frequencies and LD coefficients into Eq. (12) since they are not directly related with the SNP effects on the disease. We can extract allele frequencies from summary statistics of the GWAS and use a genotyping dataset as the reference panel for extracting the LD information. Some large projects, such as the 1000 Genomes project [25] and the HapMap3 project [26], can be used to calculate the LD coefficients. We will provide the details of calculations in Section 4.4.

In Eq. (13), the allele frequency differences $\delta_{m}(m=1,\ldots ,M)$ are critical. One may think to directly plugging in the observed allele frequencies ${\overset{ˆ}{f}}_{0,m}$ and ${\overset{ˆ}{f}}_{1,m}$ from GWAS for building the PRS model to obtain $\delta_{m}$. However, the allele frequency differences of SNPs that exhibit large effects tend to be overestimated, and these SNPs have larger contributions to PRS than the SNPs showing small effects, a phenomenon known as overfitting in the machine learning community [22]. Overestimating the SNP effects would lead to an inflated value of the predicted AUC and the incorrectly selected values of the hyperparameters. Here we adopt an empirical Bayes method to reduce the influence of overfitting by shrinking the observed allele frequency differences obtained from the summary statistics of the training GWAS.

In GWAS, we usually use the z-score calculated from the allele frequency difference test to assess the association of each SNP with the disease. Since z-scores are standardized values following a standard normal distribution N(0,1) under the null hypothesis, we will use z-scores as surrogates to derive the posterior distribution of $\delta_{m}$. The z-score is calculated with the following formula:

(15) $$z_{m}=\frac{{\overset{ˆ}{f}}_{1,m}-{\overset{ˆ}{f}}_{0,m}}{\sqrt{s_{1,m}^{2}/4n_{1}+s_{0,m}^{2}/4n_{0}}},$$

where ${\overset{ˆ}{f}}_{j,m}$ is the observed allele frequencies among controls or cases, and $s_{j,m}^{2}$ is the variance of genotypes in each group. We use $n_{0}$ and $n_{1}$ to respectively denote the sample sizes of controls and cases in the GWAS. To simplify the expression, we use $s_{m}$ to denote the denominator of the z-score, i.e.,

(16) $$s_{m}:=\sqrt{s_{1,m}^{2}/4n_{1}+s_{0,m}^{2}/4n_{0},}$$

and denote $s=\left(s_{1},\ldots ,s_{M}\right)$. We use z to encode the z-scores of all the preselected SNPs.

Based on this definition, we have $z_{m}|\delta_{m}∼N\left(\delta_{m}/s_{m},\;1\right)$ given the allele frequency difference $\delta_{m}$. We will further prove that $\delta =\left(\delta_{1},\ldots ,\delta_{M}\right)$ is actually related to the LD among the pre-selected SNPs and the underlying effects of the risk SNPs in terms of changing allele frequencies between two groups. We denote the effect of SNP m as $\beta_{m}$ and $\beta =\left(\beta_{1},\ldots ,\beta_{M}\right)$. If the SNP has no effect on the disease, then $\beta_{m}=0$. For the risk ones, $\beta_{m}\neq 0$. We assume that the standardized effect $\beta_{m}/s_{m}$ follows a point-normal distribution, i.e.,

(17) $$\frac{\beta_{m}}{s_{m}}\overset{iid}{∼}\left(1-\pi \right)\delta_{0}+\pi N\left(0,\sigma^{2}\right).$$

Here $\delta_{0}$ is a point mass at zero and π represents the prior proportion of the SNPs having effects on the disease. We use $\sigma^{2}$ to denote the variance of $\beta_{m}/s_{m}$ in the risk SNPs.

In the following two subsections, we will prove the relationship between $\delta_{m}$ and $\beta_{m}$ in two different scenarios and demonstrate how the empirical Bayes theory can help us estimate allele frequency differences by reducing the effect of overfitting.

### 4.2 Estimating AUC on independent SNPs

First, we consider the situation in which the pre-selected SNPs used for constructing PRS are independent. For example, the pre-selected SNPs in P+T are approximately independent because they are selected after an LD pruning step.

In this scenario, we have δ=β and the joint distribution of z-scores follows a multivariate normal distribution with the covariance matrix equaling to the identity matrix $I_{M}$, i.e.,

(18) $$z|\beta ∼N_{M}\left(S^{-1}\beta ,I_{M}\right),$$

where S=diag(s) is a diagonal matrix with diagonal elements encoding the standard errors of the observed allele frequency differences.

With the point-normal prior (17) on each entry of β, the log-likelihood of the z-scores is the summation of the log-likelihood for each individual z-score, i.e.

(19) $$\text{log}\;P\left(z|\pi ,\;\sigma^{2}\right)=\sum_{m=1}^{M}\text{log}\;P\left(z_{m}|\pi ,\;\sigma^{2}\right).$$

With this property, we can use an EM algorithm to get estimates of π and $\sigma^{2}$ by maximizing the likelihood $P\left(z|\pi ,\sigma^{2}\right)$.

After getting estimates of parameters π and $\sigma^{2}$, we can derive a closed-form solution for the posterior distribution of $\delta_{m}$:

(20) $$\delta_{m}|z_{m}∼\left(1-h_{m}\right)\delta_{0}+h_{m}N\left(\lambda z_{m}s_{m},\;\lambda s_{m}^{2}\right),$$

where

(21) $$h_{m}=\frac{\frac{\pi }{\sqrt{1+\sigma^{2}}}\phi \left(z_{m}/\sqrt{1+\sigma^{2}}\right)}{\left(1-\pi \right)\phi \left(z_{m}\right)+\frac{\pi }{\sqrt{1+\sigma^{2}}}\phi \left(z_{m}/\sqrt{1+\sigma^{2}}\right)}\;\text{and}\;\lambda =\frac{1}{1+1/\sigma^{2}}.$$

Here ϕ(⋅) is the probability density function of a standard normal distribution N(0,1). Derivation details of this posterior distribution can be found in the Supplementary Note. With Eq. (20), we get MC samples of $\delta_{m}|z_{m}$ and plug them as the allele frequency difference in Eq. (13) for calculating the posterior distribution of AUC. The shrink estimator of $\delta_{m}$ in (20) reduces the effect of overfitting. Details of the EM algorithm for estimating π, $\sigma^{2}$, $\delta_{m}$, and AUC are summarized in Algorithm 1.

**Algorithm 1:** Estimate AUC on independent SNPs

**Input:** z-scores $z=\left(z_{1},\ldots ,z_{M}\right)$	
**Output:** Estimated π, $\sigma^{2}$, δ and AUC	
1:	Initialize π and $\sigma^{2};$
2:	**repeat**
3:	**for** m=1,2,…,M **do**
4:	E step:
5:	$h_{m}\leftarrow \frac{\pi \phi (z_{m}/\sqrt{1+\sigma^{2}}(/\sqrt{1+\sigma^{2}}}{(1-\pi )\phi (z_{m})+\pi \phi (z_{m}/\sqrt{1+\sigma^{2}})/\sqrt{1+\sigma^{2}}}$
6:	M step:
7:	$\pi \leftarrow \frac{\sum_{m=1}^{M}h_{m}}{M}$
8:	$\sigma^{2}\leftarrow \frac{\sum_{m=1}^{M}h_{m}z_{m}^{2}}{\sum_{m=1}^{M}h_{m}}-1$
9:	**end for**
10:	**until** π and $\sigma^{2}$ converge
11:	**for** m=1,2,…,M **do**
12:	$\delta_{m}∼\left(1-h_{m}\right)\delta_{0}+h_{m}N\left(\frac{z_{m}s_{m}}{1+1/\sigma^{2}},\;\frac{s_{m}^{2}}{1+1/\sigma^{2}}\right)$
13:	**end for**
14:	$\Delta \leftarrow \frac{2\sum_{m=1}^{M}\omega_{m}\delta_{m}}{\sqrt{\tau_{0}^{2}+\tau_{1}^{2}}}$ and AUC←Φ(Δ)

### 4.3 Estimating AUC on SNPs linked by LD

When the pre-selected SNPs are not filtered by the independence criterion, their genotypes may be correlated due to LD. We can estimate the LD matrix R from a publicly available genotyping reference panel.

In this scenario, we have $\delta =SRS^{-1}\beta$ and the conditional joint distribution of the z-scores given β is

(22) $$z|\beta ∼N\left(RS^{-1}\beta ,\;R\right),$$

where S=diag(s) is a diagonal matrix encoding the standard errors of observed allele frequency differences. The proof of this property is provided in the Supplementary Note.

We used the same point-normal prior (17) on each entry of β as we used in the independent SNP scenario. There are two unknown parameters π and $\sigma^{2}$ in the prior distribution. We intend to use maximum likelihood estimation (MLE) for estimating them based on the observed z-scores. However, due to the extremely high number of component combinations (i.e., $2^{M}$), the joint likelihood of z-scores $P\left(z|\pi ,\sigma^{2}\right)$ is intractable. Here we use a Gibbs-sampling-based State-Augmentation for Marginal Estimation (SAME) algorithm to get the maximizer of the likelihood in a stochastic approach [24].

Let $\gamma_{m}\in \left\{0,\;1\right\}\;(m=1,\ldots ,M)$ denote whether SNP m has an effect on the disease or not and $\gamma =\left(\gamma_{1},\ldots ,\gamma_{M}\right)$. In the SAME algorithm, instead of evaluating the original likelihood, we assess the likelihood of the augmented data $P\left(z,\;\beta ,\;\gamma |\pi ,\sigma^{2}\right)$. With flat priors on π and $\sigma^{2}$, we derive a Gibbs sampler for sampling the full parameters β, γ, π and $\sigma^{2}$ with the joint probability proportional to the augmented data likelihood. We leave the derivation details in the Supplementary Note.

By making some simple changes to the originally derived sampler, we can get another Gibbs sampler for simultaneously sampling π, $\sigma^{2}$ and D artificial replicates of the nuisance parameters $\{\beta (d),\;\gamma (d){\}}_{d=1}^{D}$, for whom the joint probability is proportional to

(23) $$q_{D}\left(\pi ,\;\sigma^{2},\;{\left\{\beta \left(d\right),\;\gamma \left(d\right)\right\}}_{d=1}^{D}|z\right)\propto \prod_{d=1}^{D}P\left(z,\;\beta \left(d\right),\;\gamma \left(d\right)|\pi ,\;\sigma^{2}\right).$$

Based on this probability, the generated replicates of {β,γ} in the sampler are conditionally independent. With this new sampler, the marginal probability of $\left(\pi ,\sigma^{2}\right)$ can be calculated by integrating/summing over all replicates of {β,γ} :

(24) $$q_{D}\left(\pi ,\;\sigma^{2}|z\right)=\int_{\beta (D)}\sum_{\gamma (D)}\cdots \int_{\beta (1)}\sum_{\gamma (1)}q_{D}\left(\pi ,\;\sigma^{2},\;{\left\{\beta (d),\;\gamma (d)\right\}}_{d=1}^{D}|z\right)d\beta (1)\ldots d\beta (D)\;\;\propto \int_{\beta (D)}\sum_{\gamma (D)}\cdots \int_{\beta (1)}\sum_{\gamma (1)}\prod_{d=1}^{D}P\left(z,\;\beta (d),\;\gamma (d)|\pi ,\;\sigma^{2}\right)d\beta (1)\ldots d\beta (D)\;\;=\prod_{d=1}^{D}\left(\int_{\beta (d)}\sum_{\gamma (d)}P\left(z,\;\beta (d),\;\gamma (d)|\pi ,\;\sigma^{2}\right)d\beta (d)\right)\;\;=P{\left(z|\pi ,\;\sigma^{2}\right)}^{D}.$$

In other words, $\left(\pi ,\sigma^{2}\right)$ is actually sampled from $q_{D}\left(\pi ,\sigma^{2}|z\right)\propto P{\left(z|\pi ,\sigma^{2}\right)}^{D}$ in the sampler. We further denote $\left(\overset{ˆ}{\pi },{\overset{ˆ}{\sigma }}^{2}\right)=arg{max}_{\left(\pi ,\sigma^{2}\right)}\;P\left(z|\pi ,\sigma^{2}\right)$ and $\left(\tilde{\pi },{\tilde{\sigma }}^{2}\right)$ as another set of parameters. If we let D increase to infinity, the relative probability of sampling $\left(\tilde{\pi },{\tilde{\sigma }}^{2}\right)$ compared to sampling $\left(\overset{ˆ}{\pi },{\overset{ˆ}{\sigma }}^{2}\right)$ will become

(25) $$\frac{q_{D}\left(\tilde{\pi },\;\;{\tilde{\sigma }}^{2}|z\right)}{q_{D}(\overset{ˆ}{\pi },{\overset{ˆ}{\sigma }}^{2}|z)}={\left(\frac{P\left(z|\tilde{\pi },\;\;{\tilde{\sigma }}^{2}\right)}{P(z|\overset{ˆ}{\pi },{\overset{ˆ}{\sigma }}^{2})}\right)}^{D}\overset{D\to \infty }{⟶}0.$$

Therefore, the sampled $\left(\pi ,\sigma^{2}\right)$ will converge to their maximum likelihood estimates $\left(\overset{ˆ}{\pi },{\overset{ˆ}{\sigma }}^{2}\right)$ in the end.

Given their estimates, the Gibbs sampler in the SAME algorithm can provide MC samples of nuisance parameters {β,γ} with probability $P\left(\beta ,\;\gamma |z,\overset{ˆ}{\pi },{\overset{ˆ}{\sigma }}^{2}\right)$. With them, we can also get the MC samples of $\delta =SRS^{-1}\beta$ and the corresponding AUC based on Eq. (13). The complete Gibbs-sampling-based SAME algorithm for estimating π, $\sigma^{2}$, $\delta_{m}$ and AUC is summarized in Algorithm 2.

### 4.4 Calculating LD from a reference panel

Algorithm 2 needs users to input the LD matrix among the pre-selected SNPs. Some projects such as the 1000 Genomes Project [25] and the HapMap 3 project [26] have released individual-level genotype data. We can use them as reference panels to extract the LD matrix. In our method, we choose the 1000 Genomes Project as our default reference panel since it has a larger sample size. Note that most PRS methods calculate weights on the SNPs genotyped in the HapMap 3 project (HM3 SNPs) because it constitutes a set of commonly used tag SNPs that are usually well-imputed in different GWAS. To extract reliable results of the LD matrix and to reduce the computational cost of Algorithm 2, we only included HM3 SNPs in the reference panel in our experiments.

**Algorithm 2:** Estimate AUC on SNPs linked by LD

**Input:** z-scores $z=\left(z_{1},\ldots ,z_{M}\right)$
**Output:** Estimated π, $\sigma^{2}$, δ and AUC
Initialize $\pi ,\;\sigma^{2},\;\gamma_{m}∼Bernoulli(\pi )$ and $\beta_{m}∼\left(1-\gamma_{m}\right)\delta_{0}+\gamma_{m}N\left(0,\sigma^{2}\right)$ for m=1…M
D←1
$\lambda \leftarrow \frac{1}{1+1/\sigma^{-2}}$
**repeat**
**for** d←1 to D **do**
**for** m←1 to M **do**
If $\gamma_{m}=0$, $\beta_{m}\leftarrow 0$
$\mu_{m}\leftarrow \lambda (z_{m}-\sum_{m^{\prime }\neq m}\;\frac{R_{mm^{\prime }}\beta_{m^{\prime }}}{s_{m^{\prime }}})$
If $\gamma_{m}=1$, sample $\beta_{m}∼N\left(s_{m}\mu_{m},\;\lambda s_{m}^{2}\right)$
$r_{m}\leftarrow \pi \sqrt{\frac{\lambda }{\sigma^{2}}}exp(\frac{\mu^{2}}{2\lambda })$
$h_{m}\leftarrow \frac{r_{m}}{\left(1-\pi +r_{m}\right.}$
Sample $\gamma_{m}∼Bernoulli\left(h_{m}\right)$
**end for**
β(d)←β and γ(d)←γ
**end for**
Sample $\pi ∼Beta\;\left(\sum_{d=1}^{D}\;\sum_{m=1}^{M}\gamma_{m}(d)+D,MD-\sum_{d=1}^{D}\;\sum_{m=1}^{M}\gamma_{m}(d)+D\right)$
Sample $\sigma^{-2}∼Gamma\left(\frac{1}{2}\sum_{d=1}^{D}\;\sum_{m=1}^{M}\gamma_{m}(d)+D,\frac{1}{2}\sum_{d=1}^{D}\;\sum_{m=1}^{d}\beta_{m}(d{)}^{2}\gamma_{m}(d)\right)$
D←D+1
**until** $\left(\pi ,\sigma^{2}\right)$ converge.
$\delta \leftarrow SRSS^{-1}\beta ,\;\Delta \leftarrow \frac{2\sum_{m=1}^{M}\omega_{m}\delta_{m}}{\sqrt{\tau_{0}^{2}+\tau_{1}^{2}}}$ and AUC←Φ(Δ)

We note that the LD coefficient between SNPs tends to decay with increasing distance between SNPs [34]. The genotypes of SNPs with a long distance are approximately independent. We use LDetect to divide the whole genome into approximately independent blocks [35]. For human genomes with European ancestry, a total of 1,703 blocks are partitioned by LDetect.

Within each partitioned block, the correlation matrix among the genotypes of SNPs needs to be estimated as an input. Many methods have been proposed to estimate SNP covariance matrix [36–38], but most of them are sensitive to the structure of the covariance matrix or the distribution of the sample data. We note that the Ledoit-Wolf estimator does not depend on the assumptions of the covariance structure or the sample data distribution [38]. In our method, we first standardized genotypes in the reference panel, then we adopted the Ledoit-Wolf estimator on the standardized genotypes to obtain the correlation matrix.

## Figures and Tables

**Table 1:** Summary of the average values of $\rho_{AUC}$ and $rd_{AUC}$ in the simulation experiments with independent SNPs. The definitions of $\rho_{AUC}$ and $rd_{AUC}$ can be found in Section 2.2. We considered P+T as the PRS method. For each sample size, 50 replications were generated in the experiment. A high value of $\rho_{AUC}$ indicates PRStuning can accurately predict the AUC on another testing data, and a low value of $rd_{AUC}$ indicates PRStuning can effectively select a parameter that achieves performance comparable to the best-performed parameter in the testing data.

n	4, 000	6, 000	8, 000	10, 000
$\rho_{\text{AUC}}$	0.976	0.988	0.993	0.996
$rd_{\text{AUC}}$	1.3%	1.0%	0.9%	0.7%

**Table 2:** Summary of the average values of $\rho_{AUC}$ and $rd_{AUC}$ in the simulation experiments with correlated SNPs for C+T. For each sample size, 20 replications were generated in the experiment. Both PRS methods have high values of $\rho_{AUC}$ and low values of $rd_{AUC}$ in all sample size settings.

n	4, 000	6, 000	8, 000	10, 000
$\rho_{\text{AUC}}$	0.852	0.970	0.992	0.992
$rd_{\text{AUC}}$	1.9%	1.8%	1.1%	1.1%

**Table 3:** Summary of the average values of $\rho_{\mathrm{AUC}}$ and $rd_{AUC}$ in the simulation experiments with correlated SNPs for LDpred. For each sample size, 20 replications were generated in the experiment. Both PRS methods have high values of $\rho_{AUC}$ and low values of $rd_{AUC}$ in all sample size settings.

n	4, 000	6, 000	8, 000	10, 000
$\rho_{\text{AUC}}$	0.903	0.983	0.995	0.995
$rd_{\text{AUC}}$	2.0%	1.7%	1.2%	1.2%

**Table 4:** The predicted AUC values for C+T with different *p*-value thresholds in the simulation experiment based on the UKBB data. We randomly selected 80% of individuals to calculate the summary statistics as training data and the rest as testing data. We used the data collected from the 1KG as the reference panel for calculating LD. The AUC estimates from PRStuning were very close to the actual AUC values obtained from the testing data. The correlation $\rho_{\text{AUC}}$ reached 0.996 and the relative difference $rd_{AUC}$ was 3.7%.

Threshold	1	5e-1	5e-2	5e-3	5e-4	5e-5	5e-6
PRStuning	0.789	0.790	0.816	0.830	0.834	0.835	0.835
Testing	0.793	0.795	0.830	0.852	0.860	0.865	0.867

**Table 5:** The predicted AUC values for LDpred with different risk SNP proportion π in the simulation experiment based on the UKBB data. We selected 80% of individuals as training data and the rest as the testing data. The data from the 1KG were used as the reference panel. The correlation $\rho_{AUC}$ was 0.998 and the relative difference $rd_{AUC}$ was 1.3%.

π	1	3e–1	1e–1	3e–2	1e–2	3e–3	1e–3	3e–4	1e–4	3e–5	1e–5

PRStuning	0.747	0.784	0.732	0.593	0.550	0.532	0.519	0.513	0.509	0.504	0.502
Testing	0.790	0.813	0.749	0.588	0.548	0.527	0.518	0.509	0.506	0.500	0.506

**Table 6:** Summary of the publicly available GWAS summary statistics used in real data applications. The sources of GWAS summary statistics, their sample sizes, and the SNP numbers are presented in the table. We also report the number of overlapping SNPs among UKBB, 1KG, and HM3. These SNPs were used in PRStuning.

Disease	Source	Sample Size	#SNPs	#Overlapping SNPs (UKBB, 1KG, HM3)
Type 2 Diabetes (T2D)	DIAGRAM [29]	$n_{0}=56,962$ $n_{1}=12,171$	1, 938, 121	718, 340
Coronary Artery Disease (CAD)	CARDIoGRAM [30]	$n_{0}=64,762$ $n_{1}=22,233$	2, 121, 277	861, 825
Inflammatory Bowel Disease (IBD)	IIBDGC [31]	$n_{0}=38,155$ $n_{1}=48,485$	4, 911, 413	952, 376

**Table 7:** Summary of $\rho_{\mathrm{AUC}}$ and $rd_{\mathrm{AUC}}$ when using PRStuning to predict AUCs for three PRS methods on three diseases. The $rd_{AUC}$ are summarized in parenthesis. Note that the standard deviations among the AUC values with different parameters were less than 0.01 for both methods when using C+T on CAD. The extremely small standard deviations of AUC contribute to the large variation of the correlation, leading to a negative $\rho_{AUC}$.

Disease	C+T	LDpred	P+T

T2D	0.514 (3.5%)	0.982 (2.2%)	0.783 (0.5%)
CAD	−0.102 (0.4%)	0.969 (7.1%)	0.753 (0.7%)
IBD	0.858 (2.1%)	0.987 (5.6%)	0.383 (1.7%)

## References

[1] Visscher Peter M, Wray Naomi R, Zhang Qian, Sklar Pamela, McCarthy Mark I, Brown Matthew A, and Yang Jian. 10 years of GWAS discovery: biology, function, and translation. The American Journal of Human Genetics, 101(1):5–22, 2017. 28686856 10.1016/j.ajhg.2017.06.005 PMC5501872

[2] Jostins Luke and Barrett Jeffrey C. Genetic risk prediction in complex disease. Human Molecular Genetics, 20(R2):R182–R188, 2011. 21873261 10.1093/hmg/ddr378 PMC3179379

[3] Khera Amit V, Chaffin Mark, Aragam Krishna G, Haas Mary E, Roselli Carolina, Choi Seung Hoan, Natarajan Pradeep, Lander Eric S, Lubitz Steven A, Ellinor Patrick T, Genome-wide polygenic scores for common diseases identify individuals with risk equivalent to monogenic mutations. Nature Genetics, 50(9):1219–1224, 2018. 30104762 10.1038/s41588-018-0183-z PMC6128408

[4] Hill William G, Goddard Michael E, and Visscher Peter M. Data and theory point to mainly additive genetic variance for complex traits. PLoS Genetics, 4(2):e1000008, 2008. 18454194 10.1371/journal.pgen.1000008 PMC2265475

[5] Song Shuang, Jiang Wei, Hou Lin, and Zhao Hongyu. Leveraging effect size distributions to improve polygenic risk scores derived from summary statistics of genome-wide association studies. PLoS Computational Biology, 16(2):e1007565, 2020. 32045423 10.1371/journal.pcbi.1007565 PMC7039528

[6] Lin Zhen, Owen Art B, and Altman Russ B. Genomic research and human subject privacy, 2004. 10.1126/science.1095019 15247459

[7] Lunshof Jeantine E, Chadwick Ruth, Vorhaus Daniel B, and Church George M. From genetic privacy to open consent. Nature Reviews Genetics, 9(5):406–411, 2008. 10.1038/nrg2360 18379574

[8] Privé Florian, Vilhjálmsson Bjarni J, Aschard Hugues, and Blum Michael GB. Making the most of clumping and thresholding for polygenic scores. The American Journal of Human Genetics, 105(6):1213–1221, 2019. 31761295 10.1016/j.ajhg.2019.11.001 PMC6904799

[9] Wray Naomi R, Goddard Michael E, and Visscher Peter M. Prediction of individual genetic risk to disease from genome-wide association studies. Genome Research, 17(10):1520–1528, 2007. 17785532 10.1101/gr.6665407 PMC1987352

[10] International Schizophrenia Consortium. Common polygenic variation contributes to risk of schizophrenia and bipolar disorder. Nature, 460(7256):748–752, 2009. 19571811 10.1038/nature08185 PMC3912837

[11] Dudbridge Frank. Power and predictive accuracy of polygenic risk scores. PLoS Genetics, 9(3):e1003348, 2013. 23555274 10.1371/journal.pgen.1003348 PMC3605113

[12] Wray Naomi R, Lee Sang Hong, Mehta Divya, Vinkhuyzen Anna AE, Dudbridge Frank, and Middeldorp Christel M. Research review: polygenic methods and their application to psychiatric traits. Journal of Child Psychology and Psychiatry, 55(10):1068–1087, 2014. 25132410 10.1111/jcpp.12295

[13] Euesden Jack, Lewis Cathryn M, and O’reilly Paul F. PRSice: polygenic risk score software. Bioinformatics, 31(9):1466–1468, 2015. 25550326 10.1093/bioinformatics/btu848 PMC4410663

[14] Chatterjee Nilanjan, Shi Jianxin, and García-Closas Montserrat. Developing and evaluating polygenic risk prediction models for stratified disease prevention. Nature Reviews Genetics, 17(7):392–406, 2016. 10.1038/nrg.2016.27 PMC6021129 27140283

[15] Vilhjálmsson Bjarni J, Yang Jian, Finucane Hilary K, Gusev Alexander, Lindström Sara, Ripke Stephan, Genovese Giulio, Loh Po-Ru, Bhatia Gaurav, Do Ron, Modeling linkage disequilibrium increases accuracy of polygenic risk scores. The American Journal of Human Genetics, 97(4):576–592, 2015. 26430803 10.1016/j.ajhg.2015.09.001 PMC4596916

[16] Lloyd-Jones Luke R, Zeng Jian, Sidorenko Julia, Yengo Loïc, Moser Gerhard, Kemper Kathryn E, Wang Huanwei, Zheng Zhili, Magi Reedik, Esko Tõnu, Improved polygenic prediction by Bayesian multiple regression on summary statistics. Nature Communications, 10(1):1–11, 2019. 10.1038/s41467-019-12653-0 PMC6841727 31704910

[17] Ge Tian, Chen Chia-Yen, Ni Yang, Feng Yen-Chen Anne, and Smoller Jordan W. Polygenic prediction via Bayesian regression and continuous shrinkage priors. Nature Communications, 10(1):1–10, 2019. 10.1038/s41467-019-09718-5 PMC6467998 30992449

[18] Zhou Geyu and Zhao Hongyu. A fast and robust Bayesian nonparametric method for prediction of complex traits using summary statistics. PLoS Genetics, 17(7):e1009697, 2021. 34310601 10.1371/journal.pgen.1009697 PMC8341714

[19] Leonenko Ganna, Baker Emily, Stevenson-Hoare Joshua, Sierksma Annerieke, Fiers Mark, Williams Julie, Strooper Bart de, and Escott-Price Valentina. Identifying individuals with high risk of Alzheimer’s disease using polygenic risk scores. Nature Communications, 12(1):1–10, 2021. 10.1038/s41467-021-24082-z PMC8302739 34301930

[20] Machiela Mitchell J, Chen Chia-Yen, Chen Constance, Chanock Stephen J, Hunter David J, and Kraft Peter. Evaluation of polygenic risk scores for predicting breast and prostate cancer risk. Genetic Epidemiology, 35(6):506–514, 2011. 21618606 10.1002/gepi.20600 PMC4089860

[21] Song Lei, Liu Aiyi, Shi Jianxin, and Molecular Genetics of Schizophrenia Consortium Gejman PV, Sanders AR, Duan J, Cloninger CR, Svrakic DM, Buccola NG, Levinson DF, Mowry BJ, Freedman R, Olincy A, Amin F, Black DW, Silverman JM, Byerley WF. SummaryAUC: a tool for evaluating the performance of polygenic risk prediction models in validation datasets with only summary level statistics. Bioinformatics, 35(20):4038–4044, 2019. 30911754 10.1093/bioinformatics/btz176 PMC6931355

[22] Subramanian Jyothi and Simon Richard. Overfitting in prediction models-is it a problem only in high dimensions? Contemporary Clinical Trials, 36(2):636–641, 2013. 23811117 10.1016/j.cct.2013.06.011

[23] Jiang Wei and Yu Weichuan. Power estimation and sample size determination for replication studies of genome-wide association studies. BMC Genomics, 17(1):19–32, 2016. 26818952 10.1186/s12864-015-2296-4 PMC4895704

[24] Doucet Arnaud, Godsill Simon J, and Robert Christian P. Marginal maximum a posteriori estimation using Markov chain Monte Carlo. Statistics and Computing, 12(1):77–84, 2002.

[25] 1000 Genomes Project Consortium. A global reference for human genetic variation. Nature, 526(7571):68, 2015. 26432245 10.1038/nature15393 PMC4750478

[26] International HapMap 3 Consortium Integrating common and rare genetic variation in diverse human populations. Nature, 467(7311):52, 2010. 20811451 10.1038/nature09298 PMC3173859

[27] Jiang Wei, Song Shuang, Hou Lin, and Zhao Hongyu. A set of efficient methods to generate high-dimensional binary data with specified correlation structures. The American Statistician, 75(3):310–322, 2021.

[28] Bycroft Clare, Freeman Colin, Petkova Desislava, Band Gavin, Elliott Lloyd T, Sharp Kevin, Motyer Allan, Vukcevic Damjan, Delaneau Olivier, O’Connell Jared, The UK Biobank resource with deep phenotyping and genomic data. Nature, 562(7726):203–209, 2018. 30305743 10.1038/s41586-018-0579-z PMC6786975

[29] The DIAGRAM consortium. Large-scale association analysis provides insights into the genetic architecture and pathophysiology of type 2 diabetes. Nature Genetics, 44(9):981–990, 2012. 22885922 10.1038/ng.2383 PMC3442244

[30] Schunkert Heribert, König Inke R, Kathiresan Sekar, Reilly Muredach P, Assimes Themistocles L, Holm Hilma, Preuss Michael, Stewart Alexandre FR, Barbalic Maja, Gieger Christian, Large-scale association analysis identifies 13 new susceptibility loci for coronary artery disease. Nature Genetics, 43(4):333–338, 2011. 21378990 10.1038/ng.784 PMC3119261

[31] Jostins Luke, Ripke Stephan, Weersma Rinse K, Duerr Richard H, McGovern Dermot P, Hui Ken Y, Lee James C, Schumm L Philip, Sharma Yashoda, Anderson Carl A, Host-microbe interactions have shaped the genetic architecture of inflammatory bowel disease. Nature, 491(7422):119–124, 2012. 23128233 10.1038/nature11582 PMC3491803

[32] Willer Cristen J, Schmidt Ellen M, Sengupta Sebanti, Peloso Gina M, Gustafsson Stefan, Kanoni Stavroula, Ganna Andrea, Chen Jin, Buchkovich Martin L, Mora Samia, Discovery and refinement of loci associated with lipid levels. Nature genetics, 45(11):1274, 2013. 24097068 10.1038/ng.2797 PMC3838666

[33] Hand David J. Measuring classifier performance: a coherent alternative to the area under the roc curve. Machine Learning, 77(1):103–123, 2009.

[34] Ardlie Kristin G, Kruglyak Leonid, and Seielstad Mark. Patterns of linkage disequilibrium in the human genome. Nature Reviews Genetics, 3(4):299–309, 2002. 10.1038/nrg777 11967554

[35] Berisa Tomaz and Pickrell Joseph K. Approximately independent linkage disequilibrium blocks in human populations. Bioinformatics, 32(2):283, 2016. 26395773 10.1093/bioinformatics/btv546 PMC4731402

[36] Cai Tony and Liu Weidong. Adaptive thresholding for sparse covariance matrix estimation. Journal of the American Statistical Association, 106(494):672–684, 2011.

[37] Daniels Michael J and Kass Robert E. Shrinkage estimators for covariance matrices. Biometrics, 57(4):1173–1184, 2001. 11764258 10.1111/j.0006-341x.2001.01173.x PMC2748251

[38] Ledoit Olivier and Wolf Michael. A well-conditioned estimator for large-dimensional covariance matrices. Journal of Multivariate Analysis, 88(2):365–411, 2004.

